# Supplementary material for: Conditions That Simulate the Environment of Atopic Dermatitis Enhance Susceptibility of Human Keratinocytes to Vaccinia Virus
Source: Cells. 2022 Apr 14;11(8):1337. doi: 10.3390/cells11081337 (PMC9025056; doi:10.3390/cells11081337)
Supplement: Supplementary file 1 [file cells-11-01337-s001.zip › Supplementary Figure Legends.pdf]

Supplementary Figure 1: Vaccinia virus incubated in keratinocyte conditioned media demonstrate a reduction in infectivity. Vaccinia virus was incubated overnight at 37 ºC in media removed from keratinocyte cultures (N/TERT2G or primary cells) that were differentiated for two days with Ca^{2+} containing media. The remaining amount of infectious virus was assessed by titration and plaque formation on monolayers of BSC40 cells. N/TERT2G; n = 4 experiments, PHFK; n = 7 donors. Data is presented as mean with SEM.

Supplementary Figure 2: Pyridone 6 is not toxic to keratinocytes. Differentiated and undifferentiated PHFK were exposed to increasing concentrations of Pyridone 6 for 24 hours before being assessed for viability by WST-1 assay. n = 3-4 donors. Data is presented as mean with SEM. No significant differences were detected by an ANOVA test.

Supplementary Figure 3: IL-13 diminishes expression of the keratinocyte differentiation marker keratin 10. N/TERT2G cells were treated with IL-13 (50 ng/mL) or media alone at the time of differentiation and lysates were collected 2, 3 or 4 days (D) post differentiation and tested by Western blot analysis for the marker of differentiation keratin 10 (KRT10) and normalized to GAPDH expression. A representative Western blot is shown on the left and densitometric analysis of the relative protein expression normalized to media treatment (dashed line) is shown on the right. n = 3 experiments. Data is presented as mean with SEM.
