# Supplementary figures and images for: Conditions That Simulate the Environment of Atopic Dermatitis Enhance Susceptibility of Human Keratinocytes to Vaccinia Virus
Source: Cells. 2022 Apr 14;11(8):1337. doi: 10.3390/cells11081337 (PMC9025056; doi:10.3390/cells11081337)

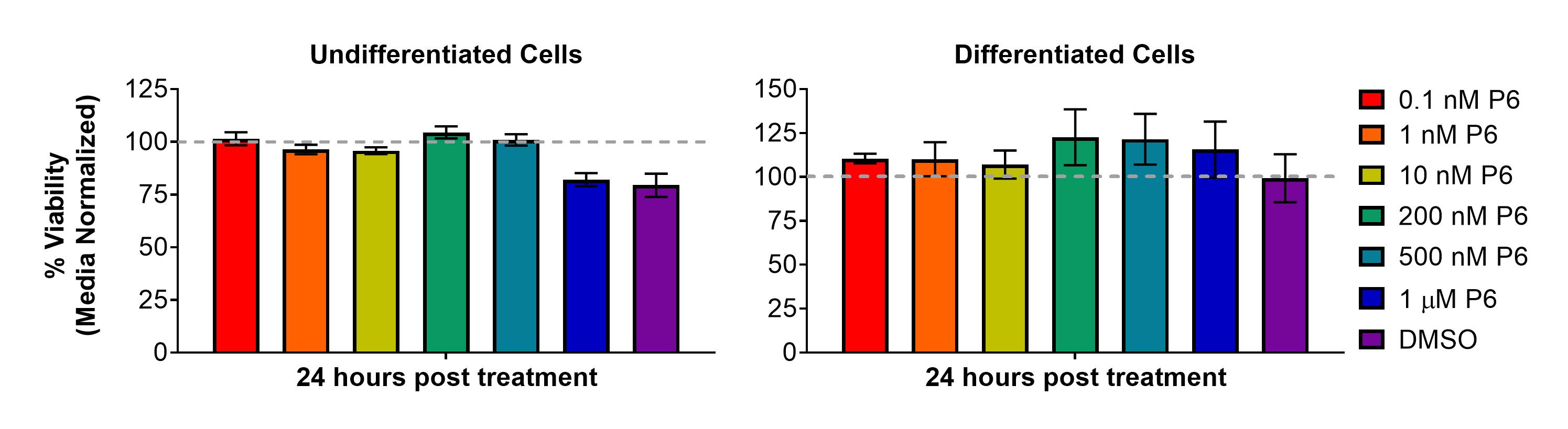

Supplement: Supplementary file 1 [file cells-11-01337-s001.zip › Fig S2.jpg]

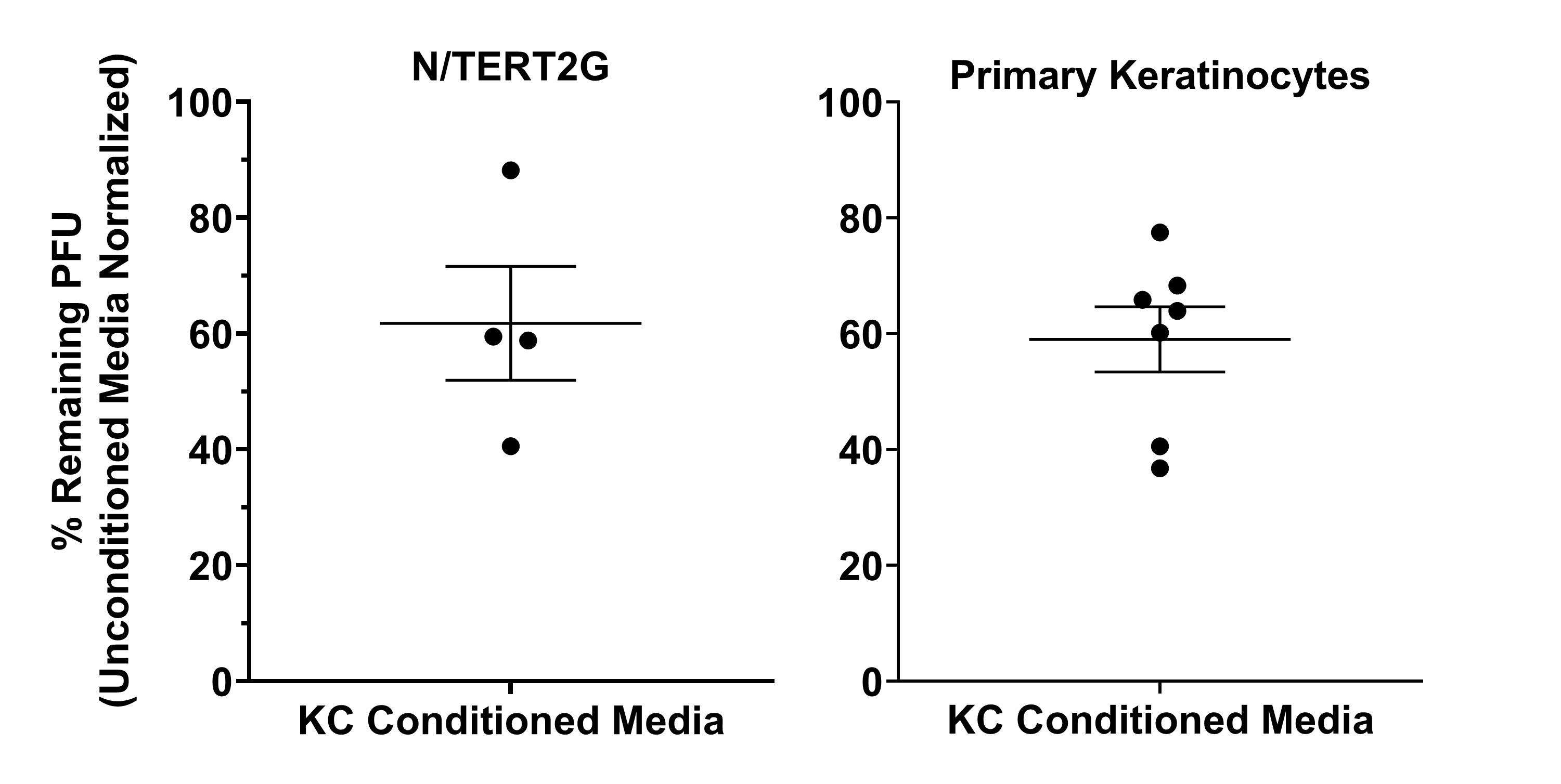

Supplement: Supplementary file 1 [file cells-11-01337-s001.zip › Figure S1.jpg]

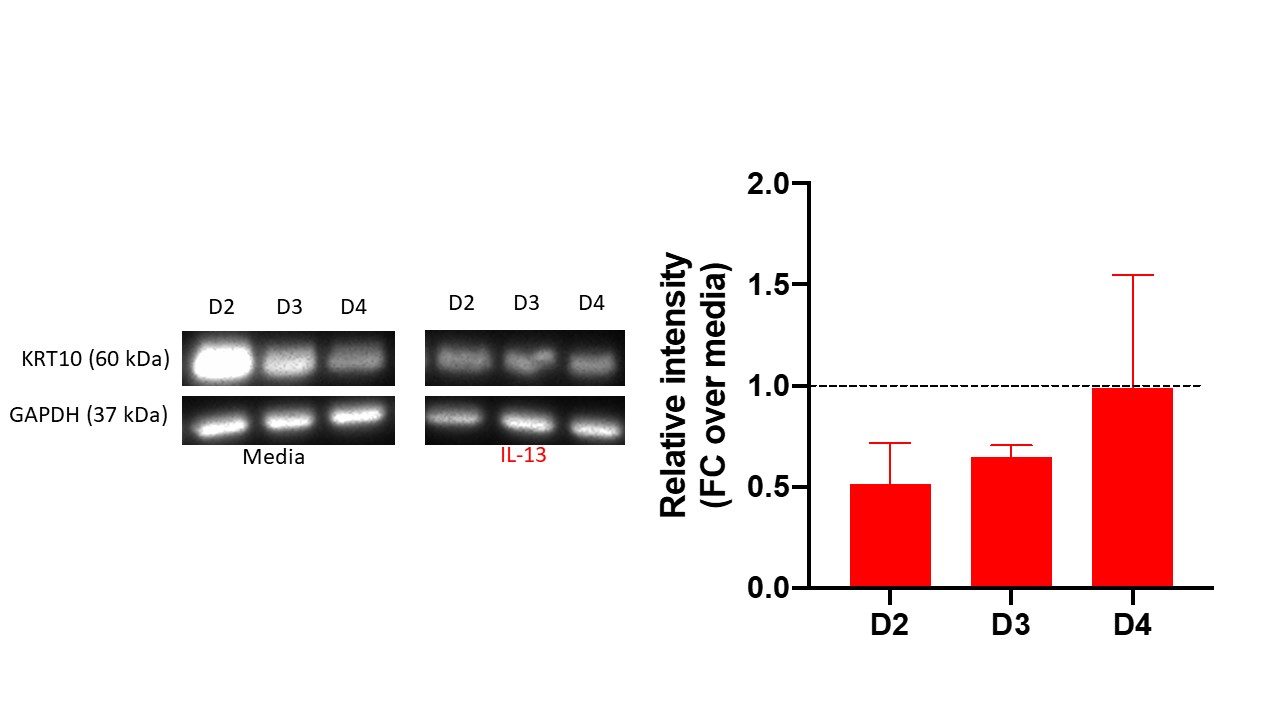

Supplement: Supplementary file 1 [file cells-11-01337-s001.zip › Figure S3.jpg]
